# The role of constraints and information gaps in driving risky medicine purchasing practices in four African countries

**DOI:** 10.1093/heapol/czae006

**Published:** 2024-02-01

**Authors:** Janelle M Wagnild, Nasima Akhter, Diana Lee, Babatunde Jayeola, Delese Mimi Darko, Moji Christianah Adeyeye, James P Komeh, David Nahamya, Adetayo Kasim, Kate Hampshire

**Affiliations:** Department of Anthropology, Durham University, South Road, Durham DH1 3LE, UK; Department of Anthropology, Durham University, South Road, Durham DH1 3LE, UK; Incidents and Substandard/Falsified Medical Products Team, World Health Organization, Avenue Appia 20, Geneva 1211, Switzerland; Incidents and Substandard/Falsified Medical Products Team, World Health Organization, Avenue Appia 20, Geneva 1211, Switzerland; Food and Drugs Authority, 17 Nelson Mandela Ave, Accra, Ghana; National Agency for Food and Drug Administration and Control, Plot 2032, Olusegun, Obasanjo Way, Zone 7, Wuse, Abuja, Nigeria; Pharmacy Board of Sierra Leone, New England Ville, Freetown, Sierra Leone; Secretary to the Authority, National Drug Authority, PO Box 23096, Kampala, Uganda; Department of Anthropology, Durham University, South Road, Durham DH1 3LE, UK; Department of Anthropology, Durham University, South Road, Durham DH1 3LE, UK

**Keywords:** Ghana, Nigeria, Sierra Leone, Uganda, medicine quality

## Abstract

Substandard and falsified (SF) medical products pose a major threat to public health and socioeconomic development, particularly in low- and middle-income countries. In response, public education campaigns have been developed to alert consumers about the risks of SF medicines and provide guidance on ‘safer’ practices, along with other demand- and supply-side measures. However, little is currently known about the potential effectiveness of such campaigns while structural constraints to accessing quality-assured medicines persist. This paper analyses survey data on medicine purchasing practices, information and constraints from four African countries (Ghana, Nigeria, Sierra Leone and Uganda; *n* > 1000 per country). Using multivariate regression and structural equation modelling, we present what we believe to be the first attempt to tease apart, statistically, the effects of an information gap vs structural constraints in driving potential public exposure to SF medicines. The analysis confirms that less privileged groups (including, variously, those in rural settlements, with low levels of formal education, not in paid employment, often women and households with a disability or long-term sickness) are disproportionately potentially exposed to SF medicines; these same demographic groups also tend to have lower levels of awareness and experience greater levels of constraint. Despite the constraints, our models suggest that public health education may have an important role to play in modifying some (but not all) risky practices. Appropriately targeted public messaging can thus be a useful part of the toolbox in the fight against SF medicines, but it can only work effectively in combination with wider-reaching reforms to address higher-level vulnerabilities in pharmaceutical supply chains in Africa and expand access to quality-assured public-sector health services.

**Key messages**

- In Ghana, Nigeria, Sierra Leone and Uganda, structurally disadvantaged groups were more likely to be potentially exposed to substandard and falsified (SF) medicines, were less likely to be aware of SF medicines and were more likely to face constraints related to medicine acquisition.
- Greater awareness of SF medicines was associated with potentially lower risk of exposure to SF medicines in Sierra Leone and Uganda, suggesting information campaigns may be useful towards mitigating some (but not necessarily all) risky practices.

## Introduction

### The global problem of substandard and falsified medical products

Substandard and falsified (SF) medical products pose a major threat to public health and socioeconomic development, particularly in low- and middle-income countries (LMICs) (WHO, 2017a; 2017b; 2019b). In terminology agreed by the World Health Assembly (2017), ‘substandard’ medical products are those that have been authorized by national authorities but fail to meet either quality standards or specifications, or both, whereas ‘falsification’ refers to the deliberate (fraudulent) misrepresentation of a drug’s identity, composition or source (Hamilton *et al.*, 2016; WHO, 2017b). In practice, the two can be difficult to distinguish but recent work suggests they may have different drivers (Pisani *et al.*, 2019). The World Health Organization (WHO) (2017b) recently estimated that over 10% of medicines in LMICs are SF, with sub-Saharan Africa particularly badly affected (Ozawa *et al.*, 2018).

SF medicines are estimated to cause more than 200 000 childhood deaths each year from malaria and pneumonia alone (Renschler *et al.*, 2015; WHO, 2017a; 2017b; Nayyar *et al.*, 2019) and to contribute significantly to antimicrobial resistance (Newton *et al.*, 2016; Brock *et al.*, 2018). The economic costs are also high, with an estimated USD30.5 billion spent each year on SF medical products in LMICs (WHO, 2019b). A recent Ugandan study suggests that both economic and health impacts are borne disproportionately by the poorest (Evans *et al.*, 2019). Experience of ineffective treatment may also undermine trust in formal healthcare systems, providers and pharmaceuticals, perhaps pushing patients towards unregulated sources (Newton *et al.*, 2010; WHO, 2017a; 2017b).

### Supply- and demand-side interventions

National governments and international organizations have sought to enhance regulatory and technical capacity in LMICs to prevent SF medicines from entering supply chains and/or intercept them before they reach consumers (Hamilton *et al.*, 2016; WHO, 2017b; Nayyar *et al.*, 2019). Prominent among these has been the Global Surveillance and Monitoring System (GSMS) launched in 2012 by the WHO, with the aim of ‘work[ing] with WHO Member States to improve the quality of reporting of substandard and falsified medical products, and, importantly, to ensure the data collected are analysed and used to influence policy, procedure and processes to protect public health, at the national, regional and the global level’ (WHO, 2017b:1; 2012). However, such efforts continue to be hampered by weak governance, the complexity and opacity of global supply chains (Mackintosh *et al.*, 2011; 2018; Tremblay, 2013) and the strong financial incentives for manufacturers and traders to cut corners, making full control of the supply side difficult to achieve.

Demand-side interventions have focused predominantly on risk communication and awareness-raising. For example, the WHO is currently launching a major global communications campaign framework (IDEAS) to inform the public of the potential risks of SF medicines and provide advice on (*inter alia*) examining medicines and packaging for abnormalities, checking manufacturers’ details and medicine expiry dates and discussing suspicions about adverse reactions with a healthcare professional (HCP) (WHO, 2017b; 2018; 2019a; 2020a). However, empowering consumers to avoid SF medical products remains challenging for at least two reasons. First, SF medical products can be very difficult to distinguish without specialist equipment, even for trained professionals. Falsified medicines are typically designed to appear identical to the genuine product and may not cause an obvious or immediate adverse reaction. The widespread availability of tableting machines, ingredients and packaging materials makes it increasingly easy to produce near-perfect copies of the original. It can be equally difficult to spot substandard medicines, particularly because poor transport or storage conditions can lead to degradation of active ingredients prior to the expiry date.

Second, following guidance requires a level of access and choice that cannot be assumed in contexts where high disease burdens, poverty and poor availability of quality-assured healthcare act as major constraints on individual agency. For the estimated two billion people in the world without effective access to essential medicines (WHO, 2020b), sourcing from ‘trusted and licensed outlets’ or seeking advice from a ‘healthcare professional’ may simply not be possible. In urgent situations (e.g. an acutely sick child), the impetus to act and ‘do something’ may further increase customers’ vulnerability to SF products (Hamill *et al.*, 2019).

### The value of public education: the current study

This situation raises important questions about the potential value of public communications campaigns for mitigating risks associated with SF medicines, especially in LMICs. In order for such a campaign to be successful, it must provide information that can be both ‘understood’ and ‘acted upon’ by the target audience within the particular social context and associated constraints (Alaszewski, 2005). We currently know very little about the relative contributions of an ‘information gap’ (potentially modifiable through a communications campaign) and structural constraints (less easily modifiable) in driving behaviours/practices^1^ that might increase exposure to SF medicines.

Drawing on survey data from four African countries (Ghana, Nigeria, Sierra Leone and Uganda), this paper has two key aims:

1. To identify, within each country, which sociodemographic groups:
  - Are more likely to engage in ‘riskier’ medicine-related practices;
  - Are less likely to be aware of, and have information about, SF medicines;
  - Are more likely to experience constraints around obtaining/using medicines.
2. To assess the relative importance of information gaps vs structural constraints in driving practices that potentially increase exposure to SF medicines.

## Background to the four countries

The four study countries were selected because all four countries have National Medicines Regulatory Authority (NMRA) focal points who have been trained to report SF medical products to the WHO GSMS, and all four countries are represented in the WHO Member State Mechanism representing widespread political will and commitment to combat this issue.

Key social, economic and health indicators for the four study countries are shown in Table 1. According to World Bank (2021) classifications, Sierra Leone and Uganda are low-income economies; Ghana and Nigeria are in the lower-middle-income bracket. There is significant variation in health indicators and healthcare provision between the four countries, though in all four countries these remain poor by global standards.

**Table 1.** Key characteristics of each country (World Bank, 2018)

Indicators	Sierra Leone	Uganda	Ghana	Nigeria	World
Population (millions)	7.7	42.7	29.8	195.9	
Rural population (% of total population)	58%	76%	44%	50%	45%
GDP per capita (USD)	528	733	2202	2230	11 433
Under five mortality (per 1000 live births)	114	48	48	120	38
Life expectancy at birth (years)	54	63	64	54	73
% of population living on <$1.90/day	43.0%	41.3%	12.7%	39.2%	9.3%
Out-of-pocket expenditure (% of all health expenditure)	45%	38%	38%	77%	18%
Physicians (per 1000 people)	0.025^a^	0.168^b^	0.136^b^	0.381	1.566^b^

a 2011 data.

b 2017, all other data refer to 2018.

Key features of each country’s medicine systems are summarized in Table 2. In each country, NMRAs are responsible for ensuring the quality and licensing of medicines across public, private and voluntary sectors, while National Medical Stores are charged with procuring and distributing medicines to public-sector facilities. However, stock-outs remain a frequent occurrence in public facilities across every country (Erhun *et al.*, 2001; Rebuild Consortium, 2011; Mukonzo *et al.*, 2013; Ahiabu *et al.*, 2018; Kahn *et al.*, 2019), requiring patients to turn to licensed private sector outlets (pharmacies and over-the-counter retailers) (Beyeler *et al.*, 2015; Ahiabu *et al.*, 2018), and sometimes to unregulated sources like market stalls and itinerant traders (Ocan *et al.*, 2014).

**Table 2.** Key features of medicine systems in Ghana, Nigeria, Sierra Leone and Uganda

	Ghana	Nigeria	Sierra Leone	Uganda
National regulators (NMRAs)	Food & Drugs Authority (FDA)	National Agency for Food and Drugs Administration and Control (NAFDAC)	Pharmacy Board of Sierra Leone	National Drugs Authority
Public procurement and supply	National (Central) and Regional Medical Stores	Federal and State Medical Stores	National Medicines Supply Agency	National Medical Stores
User fees in public-sector primary care (consultations and medicines)	Yes, unless covered by national health insurance	Yes, with exemptions for under 5s, pregnant women and over 65s in some states	Yes, with exemptions for under 5s and pregnant women/lactating mothers	No—primary care services are free of charge
National Health Insurance Scheme	Yes, covering most out-patient & in-patient services, including medication^a^	Yes, covering many services but with some exclusions^b^	Not currently functioning but in planning^c^	Not currently functioning but bill currently going through parliament^d^
Private-sector pharmacies	Licensed pharmacies (POM, P, OTC)	Licensed pharmacies (POM, P, OTC)	Licensed pharmacies (POM, P, OTC)	Licensed pharmacies (POM, P, OTC)
Other private-sector outlets licensed to sell pharmaceuticals	Licensed over-the-counter medicine shops (OTC only)	Patent & Proprietary Medicines Vendor License (OTC only)	Drugstores (some POM, P & OTC) Patent medicines shops (OTC only)	Class C Drug Shop (OTCs only)

OTC: over the counter; P: pharmacy, POM: prescription-only medicine.

All information supplied through personal communication with national regulators unless otherwise stated.

a Data from http://www.nhis.gov.gh/Default.aspx;

b Data from https://www.nhis.gov.ng/our-services/;

c Data from https://extranet.who.int/countryplanningcycles/sites/default/files/planning_cycle_repository/sierra_leone/sierra_leone_nhssp_2017-21_final_sept2017.pdf;

d Data from https://www.health.go.ug/document/proposed-national-health-insurance-scheme-bill-2019/

## Methods

### Data source

The project was developed by the WHO and NMRAs of the four countries, working through the WHO Member State Mechanism on SF medical products (WHO, 2019b; 2020a). Sample size estimation for each country to provide a representative sample at 95% confidence level for assessment of SF-related behaviours, allowing assessing a 5% difference between countries and 6–8% difference between groups (age, sex) within country, indicated that a sample of 800 would be adequate. Considering the non-response rate, distribution of age groups and feasibility of data collection across countries, an adjusted sample of 1000 was considered for each country. Data were collected by Ipsos MORI in February 2020, from a total of 4197 adults aged ≥18 years in Ghana, Nigeria, Sierra Leone and Uganda. Ethical approval was secured in advance from relevant in-country review boards and the WHO; Durham University provided ethical approval for the analysis.

In each country, questionnaires were administered via structured face-to-face interviews to a sample of adults recruited using multistage clustered random sampling who confirmed their consent before participating. Within each country, the sample was first stratified by region and by degree of urbanization (population density) based on the most recent census. Following stratification, Local Government Areas (LGAs) formed the primary sampling units (PSUs), which were randomly selected with probability proportional to the adult population (≥18 years). Within each PSU, a random starting point was selected, and *n*th household was then interviewed until the desired number of households was completed. Within a household, the eligible participant for interviewing was selected using a grid approach. Additional details about the sampling distribution are shown in Supplementary File 1. Interviews were conducted in local languages (see Supplementary file 1) and lasted approximately 10 minutes, covering topics including: sources of medicines, medicine-checking practices, information/advice received, knowledge/perceptions of SF medicines, constraints in obtaining medicines and communication/media use (see Supplementary file 2). A post-sample weighting was applied to data from each country to take account of variable distribution of age, sex and rural/urban areas so that it will approximate the distribution of the population in each country available from census data.

### Variables of interest

From the survey responses, four categories of variables were constructed: sociodemographic characteristics, which served as predictor variables in all analyses (Group A); medicine-related practices potentially associated with exposure to SF medicines (Group B); information gaps and awareness of SF medicines (Group C); and constraints related to obtaining/using medicines (Group D).

Sociodemographic predictors (Group A) included nine participant characteristics. Age (18–24, 25–34, 35–44 and ≥45 years) and gender were self-reported; location (rural/urban) was recorded by the scripter based on the pre-defined urban/rural classification of each LGA determined by the survey management team. Highest level of education was reported based on country-specific qualification scales, recoded as low/no education vs secondary/higher education. Employment status was categorized as: in paid work, in education and not in paid work or education^2^. Frequency of medicine use/purchase was classified as less than monthly vs monthly or more. Presence of disability or long-term illness in the household (including the respondent, if applicable) and presence of children (<16 years old) in the household were classified as any vs none. Finally, household size, originally reported on a continuous scale, was classified as 1–2, 3–4 or 5+. The latter two variables (children and household size) were considered as control variables because the mechanisms by which these might influence practices aside from other factors already captured in the dataset (e.g. socioeconomic status, urban/rural location, frequent medicine use) are unclear. The effects of these variables are therefore shown in the results but are not described further. Age was also included in the model as a categorical variable to control for potential confounding effects.

The main outcome set (Group B) comprised five binary variables indicating practices potentially associated with exposure to SF medicines (WHO, 2018). Three of these relate to medicine-checking practices: (1) examining expiry dates; (2) other ‘direct’ checks (including visual inspections of medicines and packaging, looking up manufacturers’ websites, etc.); and (3) asking an HCP to check medicines on their behalf. The other two variables in this group relate to purchasing practices: (4) obtaining medicines within the past 12 months from an unofficial source (general/grocery store, street hawker/market stall, friends/family or online)^3^; and (5) getting medicines without an accompanying information leaflet or label.

Eight binary variables represented indicators of awareness and information regarding SF medicines (Group C). Awareness was assessed based on whether respondents reported having heard that some medicines may not be genuine or of good quality. Respondents were also asked what, if anything, they had heard, seen or read about such medicines, including: (1) that fake or damaged medicines^4^ can cause harm, (2) that medicines should always be checked before consuming, (3) what to do if medicines seem unsafe, (4) that medicines should only be acquired from HCPs and (5) that medicines should not be bought from street hawkers or online^5^. To ascertain the likely reliability of advice about medicines, we constructed a variable indicative of whether respondents sought advice from at least one of the following: doctor, nurse, healthcare worker, pharmacist or other pharmacy staff or an appropriate charity or voluntary organization. Confidence in discussing medicines with healthcare workers was also measured, dichotomized as feeling very confident vs quite/not quite/not at all confident.

Three binary variables were used to indicate potential constraints in relation to obtaining medicines (Group D). Affordability was dichotomized as ‘big challenge or unable to afford medicines’ vs ‘small challenge or easy to afford medicines’. Medicine unavailability was determined from a yes/no question about whether the respondent had tried to obtain medicines from a pharmacy, drug store or hospital but could not because they were unavailable. Confidence in understanding instructions on how to use medicines was dichotomized as feeling very confident vs quite/not quite/not at all confident. Respondents who reported obtaining medicines from an unofficial source in the past 12 months were asked why they had done this; responses were classified as: because it was easy/simple/closest; it was the only place where medicines were known to be available; it was cheaper/free; followed a HCP’s advice; or because of lack of trust in hospitals/pharmacies.^6^ Frequencies and percentages of these variables are reported but these were not included in models because of the small size of this subgroup.

### Analyses

Analyses were restricted to respondents who indicated that they or a family member had bought or used medicines in the past 12 months. Frequencies of all predictor and outcome variables compared between countries using chi-squared analysis, accounting for sampling weights and survey design. Substantial between-country variation was observed, so all further analyses were done separately for each country. The first stage of analysis examined associations between all nine sociodemographic factors (Group A) and each outcome group: practices associated with potential exposure to SF medicines (Group B); information and awareness (Group C); and constraints (Group D); using generalized linear models (GLM) for binary outcomes with logit link. Responses of ‘don’t know’ or ‘prefer not to say’ for each variable were treated as missing. Available case analysis was performed (i.e. including all cases with responses for each variable in a given model^7^) on weighted data adjusted for age, survey design and clustering^8^, using the ‘surveys’ package in R (version 4.0.3). Results of these models were presented as error-bar plots with the x-axis on the logarithmic scale so that effect sizes above and below one were visually equivalent. We interpreted the results of these analyses through an equity lens following the PROGRESS framework (O’neill *et al.*, 2014), paying particular attention to whether axes of structural disadvantage—including by place of residence, gender, education and occupation—were associated with poorer outcomes.

**Figure 1.**
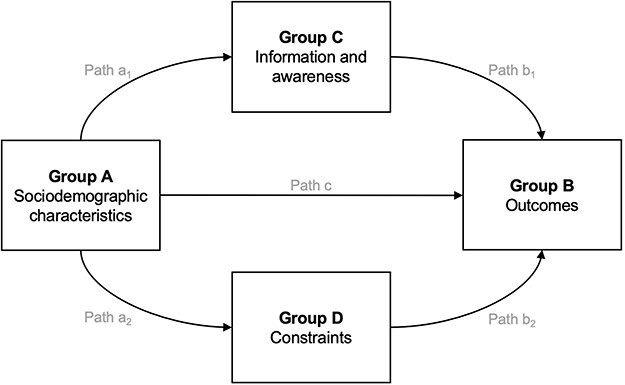
Hypothesized pathway to be tested using structural equation modelling

The second stage of analysis employed structural equation modelling (SEM) (Maruyama, 1998) to determine the relative importance of information/awareness gaps vs constraints on practices associated with potential exposure to SF medicines, whilst accounting for sociodemographic characteristics. Confirmatory latent factor analysis was performed to create four composite indices reflecting each group of variables, with variables included or excluded on the basis of goodness-of-fit criteria and model convergence. For ease of interpretation, all variables were recoded such that ‘better’ outcomes (e.g. checking expiry dates, greater awareness, greater affordability) were coded as 1 prior to loading the factors into latent constructs. The paths shown in Figure 1 were used as a starting place for constructing the SEM; from there, the model was modified iteratively until the best fit was achieved. A model was considered to fit the data well when the following criteria were met: root mean square error of approximation (RMSEA) <0.08; standardized root mean square residual (sRMR) <0.08; comparative fit index (CFI) ≥0.90; and Tucker-Lewis index (TLI) ≥0.95 (Kline, 1998; Hu and Bentler, 1999). These analyses were performed using MPlus software (version 8.5).

Findings were shared with key stakeholders via presentations during analysis and through circulations of manuscript drafts. Representatives from each NMRA are included as study co-authors.

## Results

### Description of the study participants

Of the original sample of respondents, 4129 (98% of the 4197 survey respondents) reported buying or using medicines in the preceding 12 months and comprise the analytical sample here. Sample descriptives, stratified by country, are shown in Table 3. There were significant differences between countries in terms of age, employment status, educational attainment, household size, presence of children and presence of disability or long-term illness in the household. The sample did not differ between countries in terms of gender, rural/urban location or frequency of medicine use. In all countries, the majority of respondents had low or no education, although reported educational levels were higher in Nigeria compared with the other three countries.

**Table 3.** Characteristics of the study sample (weighted frequencies)

Variables	Ghana, *n* = 1002	Nigeria, *n *= 1023	Sierra Leone, *n* = 1061	Uganda, *n* = 1043	*P*-value
**Age**					0.01
18–24	364 (41.6)	277 (27.8)	276 (25.9)	311 (29.8)	
25–34	157 (17.9)	307 (30.8)	267 (25.1)	302 (29.0)	
35–44	105 (12.0)	195 (19.5)	205 (19.2)	187 (17.9)	
45 and older	249 (28.5)	219 (21.9)	317 (29.8)	242 (23.2)	
**Gender**					0.89
Male	411 (47.0)	483 (48.5)	521 (49.0)	508 (48.8)	
Female	464 (53.0)	513 (51.5)	542 (51.0)	533 (51.2)	
**Location**					0.24
Urban	451 (51.5)	578 (58.0)	546 (51.3)	336 (32.2)	
Rural	424 (48.5)	419 (42.0)	518 (48.7)	706 (67.8)	
**Employment status**					0.002
In paid work	502 (57.4)	621 (62.3)	403 (38.4)	561 (53.9)	
In education	136 (15.5)	137 (13.7)	198 (18.9)	50 (4.8)	
Not in paid work or education	237 (27.1)	239 (24.0)	448 (42.7)	430 (41.3)	
**Education**					<0.001
Secondary or higher	122 (13.9)	415 (41.7)	204 (19.8)	78 (7.5)	
Low or none	753 (86.1)	581 (58.3)	826 (80.2)	963 (92.5)	
**Children in the household**					<0.001
None	541 (61.8)	490 (49.1)	298 (28.0)	236 (22.6)	
Any	334 (38.2)	507 (50.9)	765 (72.0)	806 (77.4)	
**Disability in household**					0.01
None	800 (91.5)	850 (85.3)	865 (81.3)	740 (71.1)	
Any	74 (8.5)	147 (14.7)	199 (18.7)	301 (28.9)	
**Household size**					<0.001
One or two	524 (59.9)	342 (34.3)	127 (11.9)	227 (21.8)	
Three to four	258 (29.5)	369 (37.0)	339 (31.9)	322 (30.9)	
Five or more	93 (10.6)	286 (28.7)	598 (56.2)	492 (47.3)	
**Frequency of medicine use**					0.05
Less than monthly	321 (37.7)	325 (33.0)	212 (20.3)	426 (41.9)	
≥ monthly	530 (62.3)	660 (67.0)	833 (79.7)	591 (58.1)	

Data shown as *n* (%).

Note: the frequencies of variables are weighted.

### Sociodemographic predictors of practices associated with potential exposure to SF medicines (Group B)

Weighted frequencies and percentages of Group B variables (practices associated with potential exposure to SF medicines) are shown by country in Table 4. There was significant between-country variation in reported levels of checking medicines. In all four countries, checking expiry dates was most widely reported, followed by other visual inspections such as checking medicine labels or batch numbers (Supplementary file 3). Prevalence of asking healthcare workers or pharmacists to check medicines did not differ between countries. Substantial proportions of respondents reported having obtained medicines from unofficial sources, ranging from 14% in Nigeria to over 30% in Uganda. The most commonly utilized unofficial sources of medicines across the four countries were street hawkers and family/friends, with smaller numbers purchasing from grocery stores, market stalls or online (Supplementary file 3).

**Table 4.** Weighted frequencies of indicators of potential exposure to SF medicines by country (Group B variables)

Country	Check medicines are not expired	Any other direct checking practices	Any asking-to-check	Bought/got from unofficial source	Medicines always have label/leaflet
Ghana	480 (54.9)	473 (54.1)	415 (47.4)	225 (26.0)	831 (95.0)
Nigeria	652 (65.4)	696 (69.8)	431 (43.2)	139 (14.1)	950 (95.3)
Sierra Leone	257 (24.2)	374 (35.2)	538 (50.6)	283 (28.6)	896 (84.2)
Uganda	558 (53.6)	494 (47.5)	426 (40.9)	301 (30.6)	841 (80.8)
*P-*value	<0.001	<0.001	0.38	0.002	<0.001

Data shown as weighted *n* (%).

**Figure 2.**
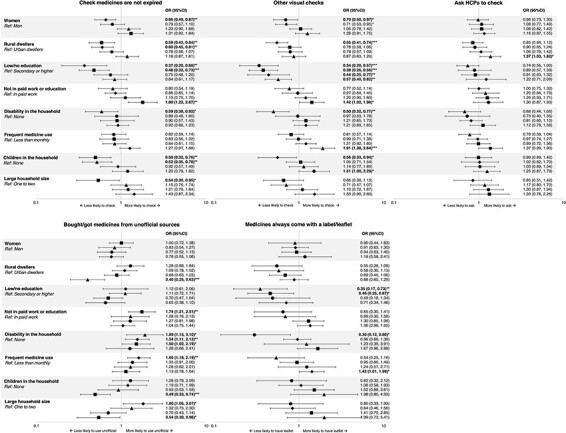
Multivariate analyses for Group B variables

Multivariate associations between sociodemographic characteristics and practices that might increase exposure to SF medicines are shown in Figure 2. Low education was the most consistent predictor, associated with lower likelihood of visual inspections in all four countries (including checking expiry dates in Sierra Leone and Uganda) and with lower likelihood getting medicines with leaflets or labels in Nigeria and Sierra Leone. Having someone in the household with a disability or long-term illness was associated with using unofficial medicine sources in all countries but Ghana; this same group was also less likely to check expiry dates or make other visual medicine checks in Nigeria and, in Ghana, was less likely to get medicines with labels/leaflets.

Gender was a significant predictor of medicine-checking practices in Uganda and Ghana, with women less likely to check expiry dates and make other visual inspections, respectively, than men. In Uganda and Sierra Leone, those living in rural areas were less likely to check medicines than their urban counterparts. However, in Nigeria, living in a rural setting was associated with lower likelihood of obtaining medicines from unofficial sources. Occupation was only significant in Sierra Leone, with those not in paid work more likely than those in paid work to report obtaining medicines from unofficial sources; however, those not in paid work were also relatively more likely to check medicines. Frequent medicine users (monthly or more often) were more likely to obtain medicines from unofficial sources (Uganda) but were also more likely to get medicines with labels/leaflets (Uganda) and to check their medicines (Nigeria).

### Sociodemographic predictors of awareness of SF medicines (Group C)

In all four countries, the vast majority of respondents were aware of the existence of non-genuine or poor-quality medicines (Table 5). Among those who had seen/heard/read anything about SF medicines, the most widely received piece of information across all countries was that ‘fake’ or ‘damaged’ medicines could cause harm. In all four countries, doctors/nurses/healthcare workers were the most common source of advice about medicines (≥70%); pharmacists were the second most common source of advice in Nigeria (69%) and Ghana (60%), while, in Sierra Leone and Uganda, it was the radio (45% and 42%, respectively) (Supplementary file 3).

**Table 5.** Frequencies and percentages of Group C variables

Country	Aware of SF medicines	SF medicines can cause harm	Should check medicines before taking them	What to do if medicines seem unsafe	Should only get medicines from HCPs	Should not get medicines from hawkers or online	Consult reliable source(s) for advice	Very confident talking about meds with HCPs
Ghana	706 (81.0)	562 (64.2)	254 (29.0)	170 (19.4)	279 (31.9)	166 (19.0)	811 (92.8)	569 (65.4)
Nigeria	905 (91.2)	657 (65.9)	310 (31.1)	230 (23.1)	388 (38.9)	252 (25.3)	967 (97.0)	702 (70.8)
Sierra Leone	788 (78.3)	767 (72.1)	203 (19.1)	176 (16.6)	390 (36.7)	170 (16.0)	869 (81.7)	642 (62.6)
Uganda	804 (77.8)	671 (64.4)	178 (17.1)	94 (9.0)	204 (19.6)	127 (12.2)	965 (92.7)	650 (62.6)
*P-*value	<0.01	0.40	0.27	0.06	0.08	0.17	<0.01	0.56

Data shown as weighted *n* (%).

**Figure 3.**
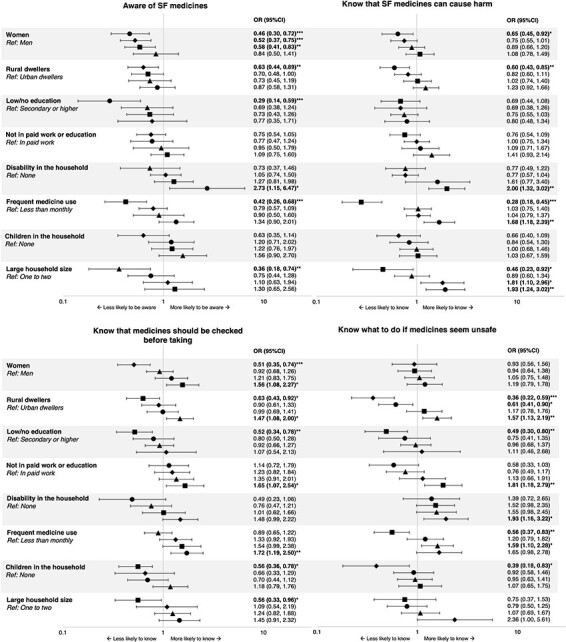

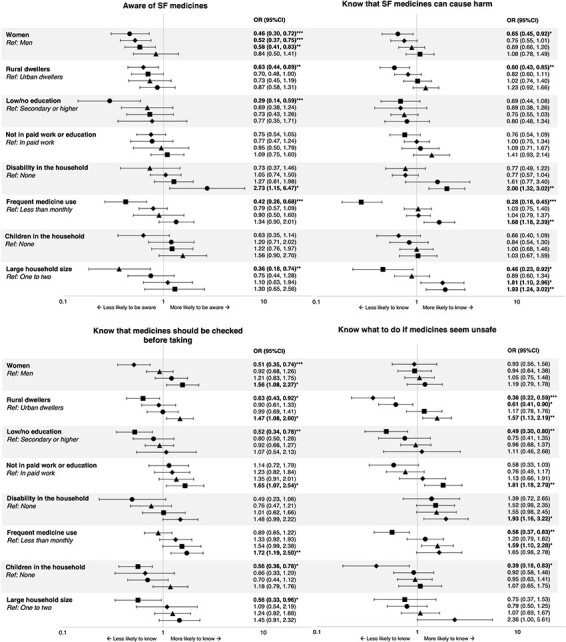

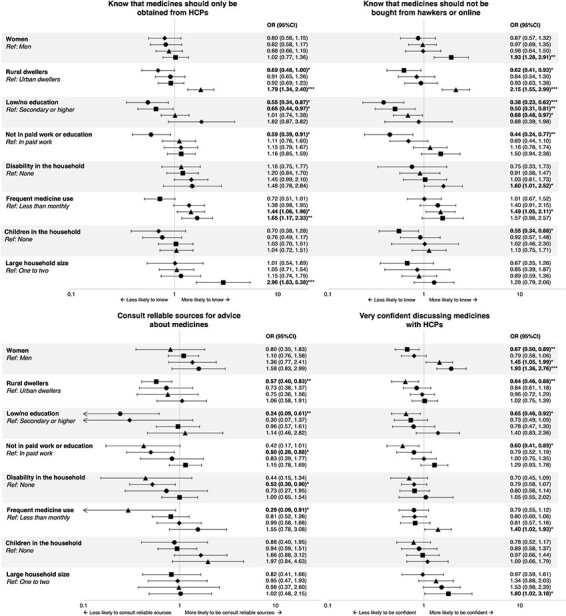
Multivariate analyses for Group C variables

Multivariate associations between sociodemographic characteristics and Group C variables are shown in Figure 3, indicating substantial variation between countries. However, two groups were generally less likely to be aware of SF medicines: those with low/no education (in all countries except Uganda) and women (all countries except Nigeria). Those with low/no education were also less likely to know that medicines should not be bought from hawkers or online (all countries except Uganda). In general, women had lower awareness about SF medicines and associated information than men. However, in Sierra Leone, women were more likely to know that medicines should not be purchased from hawkers or online and, in Nigeria and Ghana, women reported being more confident than men in discussing medicines with HCPs. In all countries except Nigeria, those with disability in the household were generally ‘more’ likely to be aware of SF medicines.

The picture is more mixed for other sociodemographic variables. Those living in rural areas tended to have lower SF medicine awareness than urban-dwellers in Ghana, Sierra Leone and Uganda; however, the opposite was true in Nigeria. In Ghana, not being in paid work was associated with lower awareness overall; however, in Sierra Leone, those not in paid work were apparently more likely to know about checking medicines and what to do if medicines seemed unsafe. Occupation was not associated with awareness in Uganda and Nigeria, although those not in paid work were less likely to consult reliable sources for advice (Uganda) and less likely to feel confident discussing medicines with HCPs (Nigeria). Finally, frequent medicine users tended to have lower awareness in Sierra Leone but higher awareness in Ghana and Nigeria. Frequent medicine use was not associated with any Group C variables in Uganda.

### Sociodemographic predictors of constraints (Group D)

Across all four countries, many respondents reported experiencing constraints in obtaining medicines, with significant variation between countries. The proportions reporting difficulty affording medicines ranged from just over 30% in Nigeria to over 60% in Sierra Leone (Table 6). Likewise, a high proportion of respondents in each country said that, within the last 12 months, they had tried to get medicines from a pharmacy, drug store or hospital but could not because they were unavailable (ranging from >30% in Sierra Leone to >60% in Uganda). In contrast, the majority of respondents in four countries (c. 60%+) claimed to feel very confident in understanding instructions on medicine use, with no significant variation by country.

**Table 6.** Frequencies and percentages of Group D outcome variables

Country	Big challenge/can’t afford medicines	Couldn’t get medicines due to unavailability	Very confident in understanding instructions on medicine use
Ghana	267 (31.2)	377 (43.1)	575 (66.0)
Nigeria	304 (30.7)	458 (45.9)	682 (69.0)
Sierra Leone	637 (61.8)	333 (31.3)	619 (60.2)
Uganda	579 (57.4)	669 (64.3)	617 (59.6)
*P-*value	<0.001	<0.001	0.43

Data shown as weighted *n* (%).

**Figure 4.**
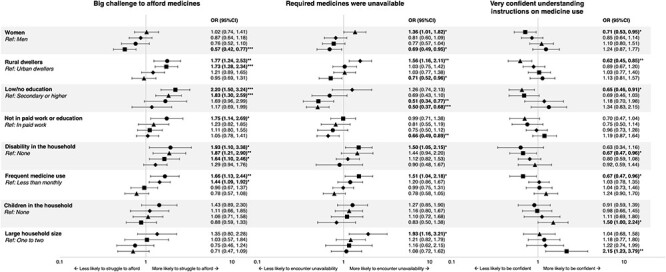
Multivariate analyses for Group D variables

Multivariate associations for Group D variables are shown in Figure 4. There was variation in the strength and direction of associations between countries. However, there was some degree of consistency in groups who reported finding it a ‘big challenge’ to afford medicines: those with a disabled person in their household (Ghana, Nigeria and Sierra Leone); those living in rural areas (in Ghana and Sierra Leone); those with lower levels of formal education (Nigeria and Sierra Leone); frequent medicine users (Ghana and Uganda); and those not in paid employment (Ghana).

The picture with encountering unavailability is rather more mixed. Those in rural areas in Uganda were more likely than their urban counterparts to report experiencing unavailability, but the opposite was true in Sierra Leone. Unexpected associations also emerged whereby those with lower levels of education (in Nigeria and Sierra Leone) and those not in paid work (Uganda) were ‘less’ likely to report problems than those with higher education or those in paid work, respectively. These discrepancies may arise from the precise wording of the question: ‘In the past 12 months, have you tried to get medicines from a pharmacy, drug store or hospital and couldn’t because they had run out or weren’t available?’ We know from other work that access to accredited medicine outlets is often more limited in rural areas (Hamill *et al.*, 2019). It is possible, therefore, that some respondents in rural areas of Sierra Leone who responded ‘no’ to that question did so because they were not able to get to a ‘pharmacy, drug store or hospital’ in the first place. Similar considerations could apply to those with lower levels formal education in Nigeria and Sierra Leone and those not in paid work in Uganda.

Responses to the ‘confidence’ question are generally in line with expectations in Sierra Leone and Nigeria. Groups reportedly less confident understanding instructions on medicine use included women (in Sierra Leone), rural dwellers (Nigeria), those with lower levels of formal education (Nigeria) and those with a disability in their household and frequent medicine users (both Sierra Leone). There were no significant associations between sociodemographic variables and ‘confidence’ in Ghana or Uganda.

Additional information on the role of constraints was available from the subgroup of respondents who had reportedly obtained medicines from unofficial sources in the preceding 12 months, who were asked to say why they had used that source (Table 7). In all four countries, the most common reason given was that it was easy/simple/closest (51–81%), suggesting that distance might be acting as a major constraint on medicine procurement practices not captured in the ‘availability’ variable. The second most reported reason was that the unofficial source was cheaper (18–45%), corroborating the finding reported above that affordability can be a significant constraint. A minority of respondents indicated they obtained medicines from unofficial sources because it was the only place they knew they were available (15–28%). Very few respondents said they got medicines from unofficial sources on the advice of an HCP (2.8–11.7%) or because they did not trust hospitals or pharmacies (0–4%).

**Table 7.** Percentages of reported reasons medicines were bought or got from unofficial sources in the past 12 months

	Ghana (n = 102)	Nigeria (n = 45)	Sierra Leone (n = 107)	Uganda (n = 120)
It was easy/simple/closest	61.8%	51.1%	81.3%	63.3%
Only known place we knew we could get medicines	19.8%	17.8%	15.0%	28.3%
Because it was cheaper than other places or free	39.6%	17.8%	30.8%	45.0%
A doctor/pharmacist/nurse told me to get it from there	9.8%	8.9%	2.8%	11.7%
Don’t trust hospitals/pharmacies	4.0%	0	0.9%	1.7%

### SEM: relative contributions of information and constraints on practices

The multivariate regression models above show that, broadly speaking, disadvantaged socio-demographic groups (women, rural dwellers, those with lower educational levels, those not in paid employment, those with a disability in the household and frequent medicine users) were (1) less well-informed about SF medicines, (2) more likely to experience constraints and (3) reported riskier practices that might increase their exposure to SF medicines, suggesting that both information and constraints might mediate the relationship between sociodemographic variables and practices. A series of SEMs was constructed to test this hypothesis and tease apart the relative contributions of information gaps and constraints.

Using the hypothesized model (Figure 1) as a starting point, we produced latent constructs that best captured the covariance between observed variables, and identified pathways for the best-fit model, based on criteria noted above. In the case of Ghana, it was not possible to produce a stable model with an acceptable level of fit to the data. For the other three countries, the Group A latent construct was best represented by the covariance between presence of children and household size; Group B comprised expiry date and other visual checks (plus not using unofficial sources in Sierra Leone); Group C contained three to five awareness measurements; and Group D comprised affordability and availability. It is worth emphasizing that it is not possible to tell whether variables within each group were excluded due to lack of covariance or redundancy (i.e. not contributing any additional information). This was of particular concern for Group B, which consists of two potentially distinctive constructs: checking practices (checking expiry dates, other visual checks, asking to check) and purchasing practices (not buying from unofficial sources, buying medicines without leaflets/labels) which may not necessarily covary. We attempted to split Group B into these separate latent constructs but convergence could not be achieved. Group B is thus presented in the SEMs as a single entity, with the note that purchasing practices may not be fully captured.

#### Nigeria

**Figure 5.**
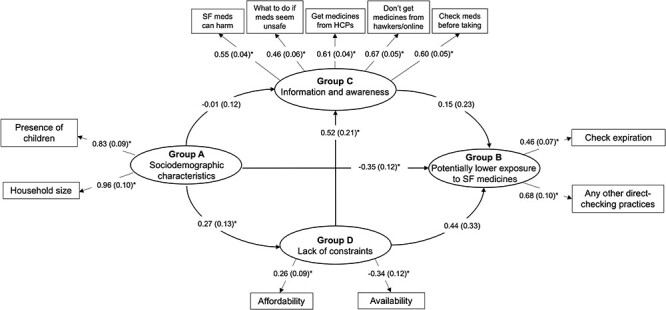
Associations between sociodemographic characteristics, information and awareness, lack of constraints and potentially lower exposure to SF medicines in Nigeria

The fit for the Nigeria SEM (Figure 5) was good (RMSEA = 0.039, sRMR = 0.053, CFI = 0.967, TLI = 0.953). In line with expectations, greater information/awareness and lack of constraints were each positively associated with lower potential exposure to SF medicines. The effect size for the impact of constraints (0.44) was larger than the effect size for the impact of awareness (0.15), although neither association was statistically significant (*P* = 0.19 and *P* = 0.51, respectively).

#### Sierra Leone

**Figure 6.**
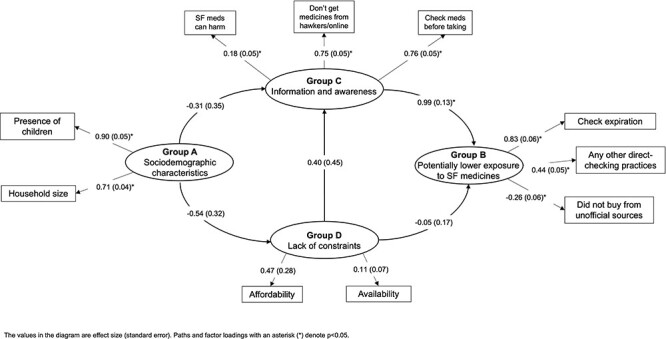
Associations between sociodemographic characteristics, information and awareness, lack of constraints and potentially lower exposure to SF medicines in Sierra Leone

For the final Sierra Leone model (Figure 6), two of the four goodness-of-fit indices indicated good fit (RMSEA = 0.065, sRMR = 0.078) but CFI and TLI were below the optimal thresholds (0.881 and 0.822, respectively), suggesting that the model may not fully capture the patterns in the data. The model indicates a positive association between information/awareness and lower potential exposure to SF medicines (*P* < 0.001). However, the link between constraints and exposure was very weak (nearly zero) and was not statistically significant (*P* = 0.76).

#### Uganda

**Figure 7.**
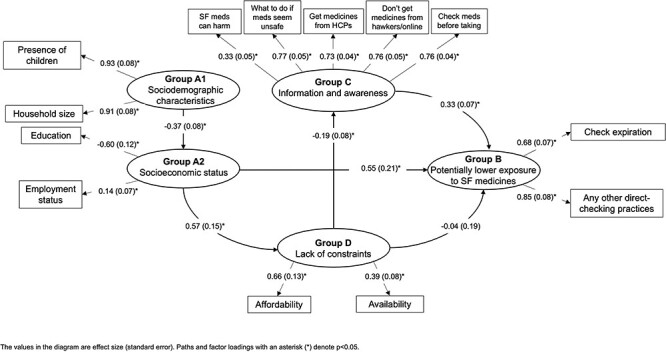
Associations between sociodemographic characteristics, information and awareness, lack of constraints and potentially lower exposure to SF medicines in Uganda

The model fit for Uganda (Figure 7) was good (RMSEA = 0.039, sRMR = 0.067, CFI = 0.972, TLI = 0.962). Again, there was a positive association between information/awareness and lower-risk practices/exposure (checking expiry date and performing other visual checks; *P* < 0.001). However, as with Sierra Leone, there was no association between constraints and lower-risk medicine practices/exposure (*P* = 0.82).

## Discussion

### Routes to potential SF medicine exposure

The first aim of this study was to determine, for each country, the association between sociodemographic variables and (1) practices associated with potential exposure to SF medicines; (2) levels of awareness/information; and (3) constraints in obtaining medicines. Findings were broadly in line with expectations: where associations were significant, they generally showed that structurally disadvantaged groups (women, rural-dwellers, those with less formal education, those not in paid work and those with a disability or long-term illness in the household) were less likely to be aware of SF medicines, more likely to experience constraints and more likely engage in practices that might elevate exposure to SF products. There was some notable variation in these associations between countries (e.g. rural-dwellers in Nigeria did not appear to suffer the same disadvantages as those in the other countries)^9^. Overall, however, it is clear that the ‘risk factors’ for lower levels of awareness, greater constraints and riskier practices broadly overlap and coincide with well-established dimensions of healthcare inequity (O’neill *et al.*, 2014).

Based on these findings, we hypothesized that the relationships between sociodemographic variables and riskier practices would be mediated through both awareness/information gaps and constraints (such as unaffordability and unavailability). SEM models constructed for Sierra Leone and Uganda suggested a link between information and medicine-related practices, such that those with greater levels of awareness of SF medicines were more likely to perform visual and other checks on medicines (a similar direction of effect was found in Nigeria but was not statistically significant). By contrast, contrary to expectations, the association between constraints and practices pathway was nearly zero in Sierra Leone and Uganda and non-significant in all models.

The fact that the SEMs for Sierra Leone and Uganda suggest a link between information/awareness and practice outcomes is encouraging. Although this cross-sectional finding cannot determine causality, it suggests that increasing awareness through appropriately targeted information campaigns could be an effective strategy for changing practices and reducing exposure to SF medicines, ‘independently of addressing underlying structural constraints’. However, an additional note of caution is important here. As noted above, the ‘Practice Group’ (B) variables contain two potentially distinctive clusters (‘checking’ practices retained in the SEMs and ‘purchasing’ practices) that may substantially differ in terms of their potential modifiability but these could not be disentangled in the SEMs. Increased public awareness might encourage people to check available medicines before purchase; however, it will not necessarily enable them to access higher-quality sources and products if these are unaffordable or otherwise out of reach. This point is supported by the multivariate regression findings which, for example, showed that those with disability/illness in the household in Uganda, Sierra Leone and Nigeria were more likely to be aware of various aspects of SF medicines, but were also more likely to use unofficial medicine sources. Taken together, this suggests that lack of awareness is only part of the story.

The absence of a significant link in the SEMs between constraints and outcomes is surprising, given the multivariate analysis results and previous work in this area. However, we would urge caution in interpreting this to mean that constraints are not relevant. As noted above, the SEMs could not effectively disentangle checking practices from purchasing practices; it is likely that the latter are more heavily impacted by constraints. It may also be that the variables available in the dataset do not capture the full range of constraints experienced on the ground. For example, as noted above, analysis of the ‘unavailability’ variable was likely limited because it effectively excluded people who were not able to reach a ‘pharmacy, drug store or hospital’ in the first place. A number of constraints identified in the literature on medicine- and care-seeking practices in these countries were not captured in this dataset; for example, distance to health facilities and associated indirect (time/transport) costs (Okeke and Okeibunor, 2010; Afari-Asiedu *et al.*, 2018; 2020; Uzochukwu *et al.*, 2018; Hamill *et al.*, 2019; Tusubira *et al.*, 2020). Analysis of responses from the subgroup who reported obtaining medicines from unofficial sources because it was easy/simple/closest further highlights the role of proximity and convenience in decision-making (Table 7). Further research that gathers more comprehensive data on constraints is required to improve our understanding of their role in driving potential exposure to SF medicines.

### Implications for policy and practice

Taken together, the findings reported here have several implications for demand-side policies and strategies aimed at reducing potential exposure to SF medicines, specifically through public communication campaigns. Notwithstanding the limitations in the data noted above and below, it is clear from our analysis that public communications campaigns ‘may’ have an important role to play in encouraging people to make basic checks of medicines before purchasing or consuming them. Such campaigns should be carefully targeted to reach the marginalized groups currently least likely to check medicines, and should take into account the different potential drivers of falsified and substandard medicines in each context (Pisani *et al.*, 2019). Depending on the country and context, these may include people living in rural areas, those with lower levels of education, those without paid employment and, in some cases, women and households that include someone with a disability or long-term sickness.

However, while raising levels of awareness and information is a positive step, this can only achieve so much on its own, for several reasons. First, as our findings might suggest, the pathway from information/awareness seems clearer for ‘checking’ than for ‘purchasing’ practices. Knowing about the risks of SF medicines may encourage people to perform visual inspections (say) but may not necessarily enable them to buy labelled medicines from official sources if these are not available or affordable to them. Second, some aspects of visual inspection (e.g. checking expiry dates, spelling mistakes) require a level of literacy—often in English or another non-local language—that cannot be assumed, especially for those with low levels of formal education. Finally, we know that SF medicines can penetrate formal supply chains and not all can be detected through visual inspection; following official advice can thus only reduce, not remove, the risk. We would also caution that raising awareness could potentially have unforeseen consequences of undermining trust in pharmaceuticals or HCPs; thus, a careful balance between communicating risk without inciting fear or mistrust is required.

### Strengths and limitations

This study is the first to our knowledge to capture in-depth quantitative data on practices, awareness and constraints surrounding medicine use in African contexts, enabling statistical analysis of associations; moreover, the use of a multistage cluster random sampling design with post-sampling weights allows the frequencies and regression findings to be nationally generalizable. However, the study also has a number of limitations. First, although a multistage cluster sampling design was used, the available data analysed did not include the specific variable defining the PSUs; standard errors and *P*-values throughout the analysis may be underestimated and should therefore be interpreted with caution. Second, the findings of the SEMs cannot be generalized as they could not account for sampling weights or the clustered study design. Third, although large samples (approximately *n* = 1000) were obtained in each country, there may not have been sufficient statistical power for all associations tested. Fourth, all outcome data were self-reported and may thus be subject to social desirability and recall bias. Fifth, as a large number of associations were tested statistically in this study, it is possible that some of the associations were spurious. Sixth, this study only took place in Anglophone countries in sub-Saharan Africa; similar studies conducted in Francophone and Lusophone African countries, as well as in Asian and South American countries that are also particularly impacted by SF medical products, are required to inform context-specific interventions that may take place there. Finally, as already noted, the survey instrument may not have adequately captured all the constraints to safer medicine procurement practices experienced in these contexts.

## Conclusion

The findings of this study have significant policy relevance for addressing demand-side drivers of SF medicine risks. First, we have shown that sociodemographic groups who are already structurally disadvantaged in access to healthcare may also be disproportionately exposed to SF medicines (as well as bearing a disproportionate share of the economic costs) (Evans *et al.*, (2019). In other words, SF medicines are not just a public health problem; they are a ‘health equity problem’. Second, those same sociodemographic groups may be less likely to be aware of SF products and more likely to experience more constraints in obtaining medicines, potentially making it difficult to act on public health advice. Third, notwithstanding those constraints, our findings suggest that appropriately targeted information campaigns may indeed have an important role to play in modifying some ‘risky’ practices, most notably encouraging people to check medicines before purchase or consumption. However, ongoing constraints on the effective agency of consumers mean that these will only be effective in combination with wider-reaching reforms to address higher-level vulnerabilities in pharmaceutical supply chains in Africa and expand access to quality-assured health services (WHO, 2017b).

## Supplementary Material

czae006_Supp

## Data Availability

The data underlying this article were provided by Ipsos MORI under licence. Data will be shared on request to the corresponding author with permission of Ipsos MORI.

## Footnotes

1. Although the term ‘behaviour change’ is widely used in public health, we prefer to talk about ‘practices’ which, as Cohn (2014) has argued, better captures ‘the emergent and contingent properties of people’s activities in particular situations’ than ‘behaviours’, which tend to be conceived of as discrete, stable, homogeneous and measurable.

2. This category includes those who were unemployed and looking for work, not working due to long-term sickness/disability, retired, looking after home/family and those reporting ‘other’ (an original response category whose interpretation is unknown).

3. There is evidence suggesting a higher prevalence of SF medicines in unofficial/unlicensed outlets than licensed outlets (see Almuzaini et al. Substandard and counterfeit medicines: a systematic review of the literature. *BMJ Open* 2013; 3:e002923).

4. This was the terminology used in the questionnaire to be more easily understandable to participants; it is synonymous with SF medicines.

5. These were free response, not prompted.

6. These were free-response, not prompted.

7. Prevalence of dropped observations due to missing responses was less than 5% for most models and never exceeded 10%.

8. The study involved a multi-stage clustered random sample design but data for primary sampling units were not available; thus, the survey design could not be fully taken into account in analyses.

9. Further discussion of between-country differences is beyond the scope of this paper, but may reflect differences in health systems and access. For example, the greater penetration of formal healthcare and medicine outlets in Nigeria may help explain the relatively low proportions of rural-dwellers in Nigeria purchasing medicines from unofficial sources.

## Reflexivity statement

As an international research project, drawing on data and insights from multiple countries, the authors on this paper are diverse in terms of regional location, seniority, gender and race/ethnic identity. The first and second authors are junior female scholars. The first three authors and the final author are all women. Five of the ten authors are based in the Global South (Ghana, Nigeria, Uganda and Sierra Leone). Eight of the authors are people of colour. Our research team is committed to inclusive global scholarship and representation.
