# Supplementary material for: The role of constraints and information gaps in driving risky medicine purchasing practices in four African countries
Source: Health Policy Plan. 2024 Feb 1;39(4):372–86. doi: 10.1093/heapol/czae006 (PMC11005838; doi:10.1093/heapol/czae006)
Supplement: czae006_Supp [file czae006_supp.zip › suppl_data/Supplementary file 1 23June.docx]

**Supplementary file 1: Sampling distributions and local languages across the four study countries**

**Table 1**: Sampling distribution across the four countries

| **Country** | **Frequency** | **Percent** |
| --- | --- | --- |
| Ghana | 1031 | 24.6 |
| Nigeria | 1025 | 24.4 |
| Sierra Leone | 1075 | 25.6 |
| Uganda | 1066 | 25.4 |
| Total | 4197 | 100.0 |

**Table 2:** Sampling distribution for Ghana

| **Region** | **Rural (n)** | **Urban (n)** | **Total (n)** |
| --- | --- | --- | --- |
| Greater Accra | 18 | 148 | 166 |
| Eastern | 60 | 49 | 109 |
| Western | 52 | 46 | 98 |
| Central | 48 | 41 | 89 |
| Brong Ahafo | 55 | 42 | 97 |
| Ashanti | 76 | 125 | 201 |
| Northern | 74 | 31 | 105 |
| Upper East | 34 | 11 | 45 |
| Upper West | 23 | 8 | 31 |
| Volta | 60 | 30 | 90 |
| **Total** | **500** | **531** | **1031** |

**Table 3:** Sampling distribution for Nigeria

| **Region** | **Rural (n)** | **Urban (n)** | **Total (n)** |
| --- | --- | --- | --- |
| Bauchi | 112 | 24 | 136 |
| Enugu | 21 | 93 | 114 |
| FCT Abuja | 25 | 24 | 49 |
| Kano | 175 | 98 | 273 |
| Lagos | 13 | 260 | 273 |
| Rivers | 94 | 86 | 180 |
| **Total** | **440** | **585** | **1025** |

**Table 4:** Sampling distribution for Sierra Leone

| **Region** | **Rural (n)** | **Urban (n)** | **Total (n)** |
| --- | --- | --- | --- |
| Eastern Province | 145 | 76 | 221 |
| Northern Province | 191 | 104 | 295 |
| Northern West Province | 150 | 89 | 239 |
| Southern Province | 75 | 94 | 169 |
| Western Area Province | 39 | 112 | 151 |
| **Total** | **600** | **475** | **1075** |

**Table 5:** Sampling distribution for Uganda

| **Region** | **Rural (n)** | **Urban (n)** | **Total (n)** |
| --- | --- | --- | --- |
| Kampala | 0 | 62 | 62 |
| Central1 | 81 | 30 | 111 |
| Central2 | 85 | 31 | 116 |
| East Central | 85 | 33 | 118 |
| Eastern | 113 | 41 | 154 |
| Karamoja | 21 | 14 | 35 |
| North | 70 | 34 | 104 |
| South Western | 114 | 41 | 155 |
| Western | 83 | 43 | 126 |
| West Nile | 62 | 23 | 85 |
| **Total** | **714** | **352** | **1066** |

**Table 6:** Local languages used for questionnaire

| **Country** | **Translation language** |
| --- | --- |
| Ghana | Dagbani, Ewe, Ga and Twi |
| Nigeria | Hausa, Igbo and Yoruba |
| Sierra Leone | Krio |
| Uganda | Acholi, Ateso, Japadhola, Karamojong, Langi, Luganda, Lugbara, Lugishu, Madi, Runyankole and Runyoro |
