# Supplementary material for: The role of constraints and information gaps in driving risky medicine purchasing practices in four African countries
Source: Health Policy Plan. 2024 Feb 1;39(4):372–86. doi: 10.1093/heapol/czae006 (PMC11005838; doi:10.1093/heapol/czae006)
Supplement: czae006_Supp [file czae006_supp.zip › suppl_data/Supplementary file 2 23June.docx]

**WHO Substandard and Falsified Medicines**

**Version 7**

**19-022095**

*Length of interview: 11.5 minutes (costed at 10 minutes)*

*Questionnaire sign-off:*

*Start fieldwork: tbc*

*End fieldwork:*

**I. INTRODUCTION**

*Hello. My name is … from Ipsos Research. We are doing a survey in this area about health and healthcare.*

*The survey is for the World Health Organisation and (insert the National Medicines Regulatory Agency of the country), who want to understand your knowledge and attitudes to certain subjects.*

RECRUITMENT AND INTERVIEWS ARE CONDUCTED IN PARTICIPANTS’ HOMES.

RANDOMLY SELECT PARTICIPANT IN HOUSEHOLD

READ OUT AND COMPLETE CONSENT FORM

First, we have a few questions about you and your household.

**II. SCREENER**

Base: all participants

1. [S] Region

*SCRIPTER: AUTOCODE REGION AND URBANITY BASED ON SAMPLE*

Base: all participants

1. [S]

INTERVIEWER: CODE GENDER

1. Male
2. Female
3. Other
4. Prefer not to say

Base: all participants

1. [S] What is your age?
2. 18 – 24
3. 24 – 34
4. 35 – 44
5. 45 – 54
6. 55 – 64
7. 65 – 74
8. 75 or older
9. Prefer not to say ***[INTERVIEWER: DO NOT READ OUT]***

Base: all participants

1. [O] Could you tell me how many people in total live in this household, including you?
   (IF MORE THAN ONE) And how many of those are aged under 16?

| Total: | ___________ |
| --- | --- |
| Children under 16: | ___________ |

Base: all participants

1. [S] Are you personally in paid work, or education, or doing something else?

PROBE TO BEST CODE

INTERVIEWER: CODE MAIN ACTIVITY ONLY (E.G. IF STUDENT AND IN PAID WORK, CODE WHICH THEY DO FOR MOST HOURS EACH WEEK)

1. In paid work (or away temporarily) (employee, self-employed, working for your family business)
2. In education, (not paid for by employer) even if on vacation
3. Unemployed and looking for work
4. Not working because long-term sick or disabled
5. Retired/no longer working because of old age
6. Looking after home or family
7. Other
8. Don’t know ***[INTERVIEWER: DO NOT READ OUT]***
9. Prefer not to say ***[INTERVIEWER: DO NOT READ OUT]***

Base: all participants

1. [S] What is your highest level of education?

PROBE TO BEST CODE

NIGERIA

1. No formal education
2. Primary
3. Secondary
4. Technical & vocational
5. Higher technical
6. Undergraduate (University)
7. Master's, Doctorate/PhD, Post-doctorate
8. Don’t know ***[INTERVIEWER: DO NOT READ OUT]***
9. Prefer not to say ***[INTERVIEWER: DO NOT READ OUT]***

UGANDA

1. No formal education
2. Primary Education
3. Lower Secondary - Ordinary Level
4. Upper Secondary - Advanced Level
5. Vocational/Technical
6. Undergraduate (University)
7. Master's, Doctorate/PhD, Post-doctorate
8. Don’t know ***[INTERVIEWER: DO NOT READ OUT]***
9. Prefer not to say ***[INTERVIEWER: DO NOT READ OUT]***

SIERRA LEONE

1. No formal education
2. Primary Education
3. Junior Secondary School
4. Secondary
5. Vocational Education
6. Undergraduate (University)
7. Master's, Doctorate/PhD, Post-doctorate
8. Don’t know **[INTERVIEWER: DO NOT READ OUT]**
9. Prefer not to say **[INTERVIEWER: DO NOT READ OUT]**

GHANA

1. No formal education
2. Primary School
3. Junior High School
4. Senior High School
5. Professional Health Sciences
6. First Level - Diplomas/Certificates
7. First Level - Higher National Diploma Teacher/Nursing Training
8. Undergraduate (University)
9. Master's, Doctorate/PhD, Post-doctorate
10. Don’t know **[INTERVIEWER: DO NOT READ OUT]**
11. Prefer not to say **[INTERVIEWER: DO NOT READ OUT]**

Base: all participants in multi-person households

1. [M] Do you or anyone else in your household have any long-term illness or disability?

***IF YES: Does this limit your normal day to day activities?***

NECESSARY EXPLAIN:

*By ‘long-term’ I mean anything that will last 6 months or more.*

*Normal day to day activities include things like eating, washing, walking, cooking or shopping.*

CODE ALL THAT APPLY

1. Yes, participant
2. Yes, another adult in the household
3. Yes, a child in the household
4. No, no-one in the household has a long-term illness or disability [S]
5. Don’t know ***[INTERVIEWER: DO NOT READ OUT]*** [S]
6. Prefer not to say ***[INTERVIEWER: DO NOT READ OUT]*** [S]

**A ATTITUDES AND BELIEFS**

Info1. [Info]

The next few questions are about you and your family.

There are no right or wrong answers – it’s important for you to answer honestly.

If there’s anything you don’t want to answer, just tell me.

Base: all participants

1. [SGRID] I’d like you to think about times when you or a member of your family has a health issue for which you personally would speak to a doctor or healthcare worker, or go to a pharmacy or drug store.
   When you have a health issue, how confident do you personally feel doing these things?
   How confident do you feel…..

*ROTATE ORDER OF STATEMENTS*

*Columns:*

1. Not at all confident
2. Not very confident
3. Quite confident
4. Very confident
5. Don’t know ***[INTERVIEWER: DO NOT READ OUT]***
6. Prefer not to say ***[INTERVIEWER: DO NOT READ OUT]***

*Rows:*

1. Making sure that the medicines you or your family use are safe and work properly
2. Understanding instructions on how to use medicines
3. Talking about health and medicines with a doctor, healthcare worker or pharmacist

**B SOURCES OF MEDICINES AND INFORMATION/ADVICE USED**

Base: all participants

1. [M] Have a look at this list of places. From which of them do you personally get advice about which medicines to use for you or your family?

SHOW SCREEN OR READ OUT

SELECT ALL THAT APPLY

*SCRIPTER: RANDOMISE GROUPS AND ITEMS WITHIN GROUPS*

1. Doctor, nurse or other healthcare worker
2. Pharmacist
3. Someone else working at a pharmacy/drug store
4. Someone working at a general store (e.g. a food shop)
5. Se.g. a food shop)
   or will access these and get them to the local fieldwork agenciesT ABOUT WHOomeone working at a street market stall
6. Street hawker
7. Newspaper or magazine
8. TV
9. Radio
10. Poster
11. Leaflet or booklet
12. Social media (e.g. Facebook, Twitter)
13. Health sites on the internet (e.g. NetMD)
14. Elsewhere on the internet
15. Friends or family
16. Colleagues or employer
17. Religious or community leaders
18. Charity or voluntary organisation relevant to a health problem or disability
19. Somewhere else. Please specify ... [O]
20. I have all the information I need/don’t want any more information ***[INTERVIEWER: DO NOT READ OUT]*** [S]
21. None of these ***[INTERVIEWER: DO NOT READ OUT]*** [S]
22. Don’t know ***[INTERVIEWER: DO NOT READ OUT]*** [S]
23. Prefer not to say ***[INTERVIEWER: DO NOT READ OUT]*** [S]

Base: all participants unless codes 17-20 at B1

1. [S] And which of them do you trust most to give you advice about which medicines to use for you or your family? Please choose up to three sources.

***SHOW SCREEN OR READ OUT***

***SELECT UP TO THREE SOURCES FROM THE LIST***

*SCRIPTER: SAME ORDER AS PREVIOUS QUESTION*

1. Doctor, nurse or other healthcare worker
2. Pharmacist
3. Someone else working at a pharmacy/drug store
4. Someone working at a general store (e.g. a food shop)
5. Se.g. a food shop)
   or will access these and get them to the local fieldwork agenciesT ABOUT WHOomeone working at a street market stall
6. Street hawker
7. Newspaper or magazine
8. TV
9. Radio
10. Poster
11. Leaflet or booklet
12. Social media (e.g. Facebook, Twitter)
13. Health sites on the internet (e.g. NetMD)
14. Elsewhere on the internet
15. Friends or family
16. Colleagues or employer
17. Religious or community leaders
18. Charity or voluntary organisation relevant to a health problem or disability
19. Somewhere else . Please specify ... [O]
20. I have all the information I need/don’t want any more information ***[INTERVIEWER: DO NOT READ OUT]*** [S]
21. None of these ***INTERVIEWER: DO NOT READ OUT]*** [S]
22. Don’t know ***[INTERVIEWER: DO NOT READ OUT]*** [S]
23. Prefer not to say ***[INTERVIEWER: DO NOT READ OUT]*** [S]

Base: all participants

Info2. [Info]

We now have a few questions about the medicines that you and your family use.

Base: all participants

1. [S] Over the past 12 months, how many times have each of these medicines been bought or used by you or your family?

*Columns:*

1. Every day
2. Every week
3. Every month
4. Less often than this
5. No-one in my family has bought or used these medicines in the past 12 months ***[INTERVIEWER: DO NOT READ OUT]***
6. Don’t know ***[INTERVIEWER: DO NOT READ OUT]***
7. Prefer not to say ***[INTERVIEWER: DO NOT READ OUT]***

*Rows:*

1. Medicines to prevent or treat malaria (called antimalarials)
2. Medicines to prevent or treat infections (called antibiotics)
3. Any other type of medicine

Base: all participants who ever buy/get any medicines at B3 (exclude those who say code 5 to all 3 iterations)

1. [M] Thinking about the medicines that you or your family have used over the past 12 months, where did they come from?

Please have a look at this list

SHOW SCREEN OR READ OUT – IDEALLY WE WOULD ASK FOR EACH OF THE DRUG GROUPS LISTED IN B3, BUT THIS WOULD MAKE THE INTERVIEW TOO LONG

*SCRIPTER: ROTATE FIRST TWO GROUPS AND RANDOMISE WITHIN GROUPS*

*SCRIPTER: DO NOT SHOW GROUP LABELS, FOR INFO ONLY*

| *Category* | *Sources* |
| --- | --- |
| **OFFICIAL SOURCES** | 1. Pharmacy 2. Drug store 3. Direct from a doctor or nurse 4. Hospital 5. Community health centre /health post or maternal and child health post [SCRIPTER: only show if country = Sierra Leone] 6. Village Health Team [SCRIPTER: only show if country = Uganda] 7. Health centre run by a nurse, clinical officer or doctor [SCRIPTER: only show if country = Uganda] |
| **UNOFFICIAL SOURCES** | 1. A shop (e.g. grocery store) 2. Market Stall 3. Street hawker 4. Bought online from a website/internet 5. Given by/sold by a family member/friend 6. Someone else. Please specify ... [FIXED POSITION] |
| **REFUSAL** | 1. Don’t know ***[INTERVIEWER: DO NOT READ OUT]*** [S] 2. Prefer not to say ***[INTERVIEWER: DO NOT READ OUT]*** [S] |

Base: all participants who select at least one unofficial source at B4

1. [M] You said that medicines were bought or got from: [INSERT UNOFFICIAL SOURCES MENTIONED: a shop, street seller or market, a website/internet, a family member/friend, someone else]. Why were they bought or got from there?

INTERVIEWER: DO NOT READ OUT – CODE PARTICIPANT’S ANSWER(S) TO THE LIST BELOW OR SELECT OTHER IF NO CODE MATCHES

SELECT ALL THAT APPLY

1. It was easy/simple/closest
2. It was the only place where I/we could get the medicines needed
3. I/we didn’t know I/we could get it somewhere else
4. A doctor/pharmacist/nurse told me to get it from there
5. It was cheaper than other places
6. It was free
7. I was/we were sure that the medicines I/we wanted would be safe from this source
8. I/we don’t trust hospitals/pharmacies
9. Another reason (specify)
10. Don’t know ***[INTERVIEWER: DO NOT READ OUT]*** [S]
11. Prefer not to say ***[INTERVIEWER: DO NOT READ OUT]*** [S]

Base: all participants who ever buy/get any medicines at B3 (exclude those who say code 5 to all 3 iterations)

1. [S] Do the medicines you or your family buy or get usually come with a label or leaflet showing what they are and how to take and store them?
2. Yes
3. No
4. Don’t know
5. Prefer not to say

Base: all participants who ever buy/get any medicines at B3 (exclude those who say code 5 to all 3 iterations)

1. [S] And thinking about the money that is spent buying medicines for you or your family, which of these best describes your situation?

SHOW SCREEN OR READ OUT

*SCRIPTER: FLIP SCALE (1-4) FOR ALTERNATE INTERVIEWS*

1. I/we can **easily afford to** pay for all the medicines we need
2. It’s a **small challenge** to pay for all the medicines we family need
3. It’s a **big challenge** to pay for all the medicines we need
4. I/we **can’t afford** to pay for all the medicines we need
5. Not applicable, all the medicines needed are free
6. Don’t know ***[INTERVIEWER: DO NOT READ OUT]***
7. Prefer not to say ***[INTERVIEWER: DO NOT READ OUT]***

Base: all participants

1. [O] When getting medicines for you or your family, what do you do to make sure they are safe and work properly?

INTERVIEWER: PROBE FULLY, RECORD ANSWERS VERBATIM:

PROBE: What do you mean by that? Could you explain that? What exactly do you do/look for? What else?

|  |
| --- |

Base: all participants

1. [M] Please look at this list. Which, if any, of these things are done to check that medicines for you or your family are safe and work properly?

INTERVIEWER: SHOW SCREEN OR READ OUT

SCRIPTER: ROTATE WITHIN BLOCKS (OTHER ANSWERS – CODES 6 AND 11) ALWAYS AT THE BOTTOM OF THE BLOCK

SELECT ALL THAT APPLY

Find out information

1. Ask a healthcare worker (doctor, nurse) to check
2. Ask a pharmacist to check
3. Look up the medicines on the manufacturer’s/brand’s website
4. Look up the medicines elsewhere online/on the internet
5. Ask a friend, colleague, family member to check the medicines (someone who is not a healthcare worker)
6. Check with someone else (specify who)

Other actions

1. Only get medicines from a healthcare worker or pharmacist
2. Make sure a receipt is given for the medicines
3. Only get medicines that have a label or information showing what they are and how to take or store them
4. Check that the medicines are real/genuine (e.g. check labels, batch numbers)
5. Chow screen or read out
   edicines for you or your family are safe and effective? nuine and good quality. We have heck that the medicines are not too old/out of date/expired
6. Take some other action (specify)
7. None of these ***[INTERVIEWER: DO NOT READ OUT]*** [S]
8. Don’t know ***[INTERVIEWER: DO NOT READ OUT]*** [S]
9. Prefer not to say ***[INTERVIEWER: DO NOT READ OUT]*** [S]

**C PERCEPTIONS OF DANGERS OF TAKING FALSIFIED/SUBSTANDARD MEDICINES**

Base: all participants

1. [S] Have you personally heard that there may be medicines available which are not genuine and good quality. They could be fake and do not treat the disease as they should, or damaged for example too old, expired, or not properly stored?
2. Yes – have heard
3. No – have not heard
4. Don’t know ***[INTERVIEWER: DO NOT READ OUT]***
5. Prefer not to say ***[INTERVIEWER: DO NOT READ OUT]***

Base: all participants

1. [M] Why do you think some people might use medicines which are not genuine and good quality?

INTERVIEWER: DO NOT READ OUT – CODE PARTICIPANT’S ANSWER TO THE LIST BELOW OR SELECT OTHER IF NO CODE MATCHES

*SCRIPTER: LIST IS FIXED*

1. They don’t know the medicines are not genuine and good quality
2. They don’t know how to check that the medicines are genuine and good quality
3. They are cheaper
4. They think they will work almost as well as other medicines
5. They are easier to find than other medicines
6. They are the only medicines that are available
7. They have been recommended by a healthcare worker
8. Some other reason (specify)
9. Don’t know ***[INTERVIEWER: DO NOT READ OUT]*** [S]
10. Prefer not to say ***[INTERVIEWER: DO NOT READ OUT]*** [S]

Base: all participants

1. [M] In the past 12 months have any of these things happened to you or your family?

READ OUT. SELECT ALL THAT APPLY

1. Tried to get medicines from a pharmacy, drug store or hospital and you couldn’t because they had run out or weren’t available
2. Tried to buy medicines recommended or prescribed by a healthcare worker, but couldn’t because they were too expensive
3. Bought medicines that were different from what the healthcare worker recommended or prescribed
4. A seller said to use a different medicine because they were just as good or better than the prescription
5. A medicine didn’t work or made you/someone in your family ill
6. None of these [S]
7. Don’t know ***[INTERVIEWER: DO NOT READ OUT]*** [S]
8. Prefer not to say ***[INTERVIEWER: DO NOT READ OUT]*** [S]

Base: all participants

1. [S] For you, how easy or difficult is it for you personally to make sure that medicines you for you or your family are genuine and good quality?

SHOW SCREEN OR READ OUT

SELECT ALL THAT APPLY

1. Very difficult
2. Quite difficult
3. Quite easy
4. Very easy
5. Don’t know ***[INTERVIEWER: DO NOT READ OUT]***
6. Prefer not to say ***[INTERVIEWER: DO NOT READ OUT]***

Base: all participants

Now I’m going to ask you about things that you and your family have done recently.

It’s important that you answer honestly, and please remember that your answers are confidential.

(IF NECESSARY: This means that your answers will be kept private and will only be used for this research survey.)

If you’d prefer not to say your answers out loud, you can point to the screen to show which answer you want to give.

(IF NECESSARY, EXPLAIN THAT THE BLUE BUTTON MEANS YES AND THE YELLOW BUTTON MEANS NO)

Base: all participants

1. [S] In the past 12 months, have you or someone in your family bought medicines from people who are not medically qualified. By that, I mean buying them online, from a street hawker or market seller, or from friends or family?
2. Yes [SCRIPTER: insert blue button]
3. No [SCRIPTER: insert yellow button]
4. Don’t know
5. Don’t want to answer

Base: all participants

1. [S] In the past 12 months, have you or someone in your family bought or got medicines that you thought were not genuine and good quality?
2. Yes [SCRIPTER: insert blue button]
3. No [SCRIPTER: insert yellow button]
4. Don’t know
5. Don’t want to answer

**D COMMUNICATIONS AND MEDIA USE**

These are the last few questions.

Base: all participants

1. [M] Have you personally seen or heard or read anything recently about the dangers of using medicines which are not genuine and good quality? **If YES**, what have you seen or heard?

INTERVIEWER: DO NOT READ OUT – CODE PARTICIPANT’S ANSWER TO THE LIST BELOW OR SELECT OTHER IF NO CODE MATCHES

1. That fake or damaged medicines can harm you or your family
2. What to do if you get medicines that you think are unsafe (e.g. that you shouldn’t use them, who you should tell)
3. That you should only get or buy medicines from healthcare workers (doctors, nurses, pharmacists)
4. That you shouldn’t buy medicines from street hawkers or online
5. That you should always check the medicines that you are given before taking them (e.g. check they aren’t old, expired or damaged)
6. No – have not seen or heard or read anything ***[INTERVIEWER: DO NOT READ OUT]*** [S]
7. Don’t know ***[INTERVIEWER: DO NOT READ OUT]*** [S]
8. Prefer not to say ***[INTERVIEWER: DO NOT READ OUT]*** [S]

Base: all participants

1. [M] Which of these things, if any, have you personally done in the last few months?

SELECT ALL THAT APPLY

1. Used social network sites like Twitter or Facebook
2. Online shopping/bought things online
3. Found out about government health services online
4. Visited a website that contains information about health
5. Used a health related mobile phone app
6. Listened to a radio programme about health
7. Watched a TV programme about health
8. Received health information through SMS/text message on your phone
9. Received health information on printed material e.g. leaflet, poster, booklet, etc.
10. None of these ***[INTERVIEWER: DO NOT READ OUT]*** [S]
11. Don’t know ***[INTERVIEWER: DO NOT READ OUT]*** [S]
12. Prefer not to say ***[INTERVIEWER: DO NOT READ OUT]*** [S]

ADD:

Interviewer code language in which interview was conducted

- English
- Other language (choose from country list, or specify if other)

Interviewer code how self-completion questions were answered

- Participant answered questions by pressing button on screen for themselves
- Participant told the interviewer which button to press

**THANK YOU AND CLOSE**
