# Supplementary material for: The role of constraints and information gaps in driving risky medicine purchasing practices in four African countries
Source: Health Policy Plan. 2024 Feb 1;39(4):372–86. doi: 10.1093/heapol/czae006 (PMC11005838; doi:10.1093/heapol/czae006)
Supplement: czae006_Supp [file czae006_supp.zip › suppl_data/Supplementary file 3 23June.docx]

**Table S1.** Frequencies and percentages of medicine-checking practices.

|  | **Ghana** | | **Nigeria** | | **Sierra Leone** | | **Uganda** | | **p-value** |
| --- | --- | --- | --- | --- | --- | --- | --- | --- | --- |
| **Variables** | **n** | **%** | **n** | **%** | **n** | **%** | **n** | **%** |  |
| **Check medicines are not expired** | 480 | 54.9 | 652 | 65.4 | 257 | 24.2 | 558 | 53.6 | <0.001 |
| **Other direct checking practices** |  |  |  |  |  |  |  |  |  |
| Check that medicines are real/genuine (e.g. check labels, batch numbers) | 295 | 33.8 | 538 | 54.0 | 212 | 19.9 | 277 | 26.6 | <0.001 |
| Only get medicines that have a label or information telling me what they are and how to take or store them | 199 | 22.7 | 280 | 28.1 | 166 | 15.6 | 258 | 24.8 | 0.08 |
| Make sure a receipt is given for medicines | 90 | 10.3 | 122 | 12.2 | 33 | 3.1 | 134 | 12.9 | <0.01 |
| Look up the medicines on the manufacturer's/brand's website | 67 | 7.7 | 96 | 9.6 | 41 | 3.9 | 72 | 6.9 | 0.05 |
| **Asking-to-check practices** |  |  |  |  |  |  |  |  |  |
| Ask a healthcare worker (doctor, nurse) to check | 257 | 29.4 | 282 | 28.3 | 404 | 38.0 | 369 | 35.4 | 0.31 |
| Ask a pharmacist to check | 275 | 31.4 | 305 | 30.6 | 253 | 23.8 | 164 | 15.8 | <0.01 |
| **Other responses** |  |  |  |  |  |  |  |  |  |
| Only get medicines from a healthcare worker or pharmacist | 194 | 22.2 | 319 | 32.0 | 237 | 22.3 | 389 | 37.4 | <0.01 |
| Ask a friend, colleague, family member to check the medicines (someone who is not a healthcare worker) | 172 | 19.7 | 154 | 15.4 | 110 | 10.3 | 176 | 16.9 | 0.11 |
| Look up the medicines elsewhere online/on the internet | 48 | 5.5 | 62 | 6.2 | 14 | 1.3 | 24 | 2.3 | 0.03 |
| Take some other action | 0 | 0.0 | 9 | 0.9 | 0 | 0.0 | 0 | 0.0 | n/a |
| Nothing | 34 | 3.9 | 4 | 0.4 | 38 | 3.6 | 81 | 7.8 | <0.01 |

Data shown as weighted n (%)

**Table S2**. Frequencies and percentages of sources of medicines

|  | **Ghana** | | **Nigeria** | | **Sierra Leone** | | **Uganda** | | **p-value** |
| --- | --- | --- | --- | --- | --- | --- | --- | --- | --- |
| **Variables** | **n** | **%** | **n** | **%** | **n** | **%** | **n** | **%** |  |
| **Official sources of medicines** |  |  |  |  |  |  |  |  |  |
| Pharmacy | 579 | 66.2 | 812 | 81.4 | 738 | 69.4 | 402 | 38.6 | <0.001 |
| Hospital | 466 | 53.3 | 541 | 54.3 | 777 | 73.0 | 488 | 46.9 | 0.01 |
| Drug store | 514 | 58.7 | 493 | 49.4 | 40 | 3.8 | 465 | 44.6 | <0.001 |
| Direct from a doctor or nurse | 162 | 18.5 | 225 | 22.6 | 123 | 11.6 | 173 | 16.6 | 0.20 |
| Community health centre/health post (Sierra Leone only) |  | N/A |  | N/A | 148 | 13.9 |  | N/A | N/A |
| Health centre run by nurse, clinical officer or doctor (Uganda only) |  | N/A |  | N/A |  | N/A | 599 | 57.5 | N/A |
| Village health team (Uganda only) |  | N/A |  | N/A |  | N/A | 139 | 13.3 | N/A |
| **Unofficial sources of medicines** |  |  |  |  |  |  |  |  |  |
| Street hawker | 53 | 6.1 | 9 | 0.9 | 85 | 8.0 | 36 | 3.5 | 0.001 |
| Given by/sold by a family member/friend | 42 | 4.8 | 20 | 2.0 | 9 | 0.8 | 55 | 5.3 | 0.01 |
| Shop (e.g., grocery store) | 13 | 1.5 | 9 | 0.9 | 9 | 0.8 | 33 | 3.2 | 0.09 |
| Market stall | 7 | 0.8 | 10 | 1.0 | 7 | 0.7 | 17 | 1.6 | 0.42 |
| Bought online from a website/Internet | 4 | 0.5 | 1 | 0.1 | 2 | 0.2 | 0 | 0.0 | 0.09 |

Data shown as weighted n (%)

**Table S3**. Frequencies and percentages of sources of advice about medicines

|  | **Ghana** | | **Nigeria** | | **Sierra Leone** | | **Uganda** | | **p-value** |
| --- | --- | --- | --- | --- | --- | --- | --- | --- | --- |
| **Variables** | **n** | **%** | **n** | **%** | **n** | **%** | **n** | **%** |  |
| **Reliable sources of advice** |  |  |  |  |  |  |  |  |  |
| Doctor, nurse or other healthcare worker | 672 | 76.9 | 851 | 85.4 | 744 | 69.9 | 875 | 84.0 | 0.02 |
| Pharmacist | 526 | 60.1 | 691 | 69.4 | 357 | 33.6 | 289 | 27.8 | <0.001 |
| Someone else working at a pharmacy/drugstore | 288 | 33.0 | 300 | 30.1 | 37 | 3.5 | 338 | 32.5 | <0.001 |
| Charity or voluntary organisation relevant to a health problem or disability | 32 | 3.7 | 25 | 2.5 | 19 | 1.8 | 89 | 8.5 | <0.001 |
| **Other sources of advice** |  |  |  |  |  |  |  |  |  |
| Radio | 300 | 34.3 | 164 | 16.4 | 476 | 44.7 | 441 | 42.3 | <0.001 |
| Friends or family | 289 | 33.0 | 242 | 24.3 | 102 | 9.6 | 394 | 37.8 | <0.001 |
| TV | 367 | 42.0 | 102 | 10.2 | 112 | 10.5 | 186 | 17.9 | <0.001 |
| Religious or community leaders | 68 | 7.8 | 58 | 5.8 | 48 | 4.5 | 175 | 16.8 | <0.01 |
| Poster | 29 | 3.3 | 24 | 2.4 | 33 | 3.1 | 110 | 10.6 | <0.001 |
| Newspaper or magazine | 30 | 3.4 | 21 | 2.1 | 30 | 2.8 | 85 | 8.2 | <0.01 |
| Leaflet or booklet | 104 | 11.9 | 66 | 6.6 | 10 | 0.9 | 83 | 8.0 | <0.01 |
| Colleagues or employer | 67 | 7.7 | 27 | 2.7 | 18 | 1.7 | 74 | 7.1 | <0.001 |
| Street hawker | 39 | 4.5 | 14 | 1.4 | 16 | 1.5 | 51 | 4.9 | 0.03 |
| Social media (e.g., Facebook, Twitter) | 63 | 7.2 | 65 | 6.5 | 14 | 1.3 | 42 | 4.0 | <0.01 |
| Someone working at a general store (e.g., a food shop) | 33 | 3.8 | 18 | 1.8 | 8 | 0.8 | 42 | 4.0 | 0.03 |
| Elsewhere on the Internet | 39 | 4.5 | 25 | 2.5 | 1 | 0.1 | 33 | 3.2 | <0.01 |
| Someone working at a street market stall | 14 | 1.6 | 14 | 1.4 | 8 | 0.8 | 30 | 2.9 | 0.19 |
| Health sites on the Internet (e.g., NetMD) | 58 | 6.6 | 37 | 3.7 | 20 | 1.9 | 23 | 2.2 | 0.03 |

Data shown as weighted n (%)
