# Supplementary material for: The role of constraints and information gaps in driving risky medicine purchasing practices in four African countries
Source: Health Policy Plan. 2024 Feb 1;39(4):372–86. doi: 10.1093/heapol/czae006 (PMC11005838; doi:10.1093/heapol/czae006)
Supplement: czae006_Supp [file czae006_supp.zip › suppl_data/Response to reviewer comments.docx]

**Point-by-point response to reviewer comments**

Consumer exposure to substandard and falsified medicines in Africa: Constraints, information gaps and the potential role of risk communication

| **Comment** | **Response** |
| --- | --- |
| **Reviewer 1** |  |
| The ms has been greatly improved in response to the reviewers' suggestions. My only remaining puzzle is the response to P20, L27 - what was the statistical power? and the response 'We do not have exact information as to level of power that was used (i.e. 80% or 90%).'. Perhaps this reflects my lack of statistical knowledge but would not a power  % have been needed to be specified in order to do the sample size calculation ? Would it be possible to ask the person who performed this calculation ? If that is not possible, could be estimated from the sample size calculation given ? | In certain kinds of study designs (e.g. experimental designs), yes, the power would be specified in the calculation as it pertained to the hypothesized association between the exposure variable and the primary outcome. This does not pertain to survey sample size calculations; these require a specified confidence level (95% used here, as stated in the manuscript) along with other inputs such as estimated proportions and population size. |
|  |  |
| **Reviewer 2** |  |
| - I still feel that some of your claims are not justified by your results. You make statements like “Across the four countries, having low/no education, having someone with a disability in the household, being a woman, and living in a rural area were generally associated with riskier practices” pg 21, “structurally disadvantaged groups (women, rural-dwellers, those with less formal education, those not in paid work, and those with a disability or long-term illness in the household) tended to have lower levels of knowledge/awareness of SF medicines, experience more constraints, and engage in practices that might elevate exposure to SF products” pg 27, and “those same sociodemographic groups generally have lower levels of awareness/information about SF products and experience more constraints in obtaining medicines” pg 31. However, the majority of associations tested are not significant. As I noted in my previous review looking at Fig 2 for example, having low education is only significant in 8/20 of associations, disability in 3/20, women in 3/20 and rural in 4/20. I do not feel that making a reference to the O’Neil model is relevant to this issue – it’s about interpreting your findings. | Thank you for raising this. We have entirely removed the sentence “Across the four countries…” from (what had been) page 21. We have rephrased the sentence on (what was) page 27 specify that “*where associations were significant*, they generally showed that structurally disadvantaged groups…were less likely to be aware of SF medicines, more likely to experience constraints, and more likely to engage in practices that might elevate exposure to SF products”. We have also reworded the sentence on (what had been) page 31 to say "those same sociodemographic groups *may be* less likely to be aware of SF products and more likely to experience more constraints in obtaining medicines”.  We have retained the O’Neill reference as we think it still relevant, but we hope the above changes have clarified interpretations of our findings. |
| Thanks for adding in “potential” before exposure to SF meds in most places. I still noted that this was missing in the abstract and in the subhead 5.1. I would still suggest that you discuss in the Discussion whether there is any empirical evidence of associations between these behaviours and SF medicine exposure – I don’t think referring to a WHO fact sheet is sufficient. (Plus the https link to the WHO 2016 fact sheet  did not work for me, and I had the same problem with the WHO 2012 Resolution link.) | Thank you for spotting this – we have added ‘potential’ in the abstract and the subheading for 5.1.  There is evidence suggesting a higher prevalence of SF medicines in unofficial/unlicensed outlets than licensed outlets (see Almuzaini et al. Substandard and counterfeit medicines: a systematic review of the literature. *BMJ Open* 2013; 3:e002923); we have added this reference as a footnote in the methods (section 3.2). While there are no studies to our knowledge that have demonstrated a direct causal relationship between medicine checking practices and lower exposure to SF meds, it is reasonable to assume that checking for a damaged or expired medicine before purchase reduces the likelihood of getting a damaged or expired medicine. However, not all SF medicines can be detected through visual inspection alone (as we note in section 1.2), and therefore there are ongoing efforts to increase the efficacy of consumer checking via new low-tech authentication technologies. We have retained the reference to the WHO fact sheet because this is the current advice provided to member states.  Thank you for checking the links. We have updated the link for the 2012 Resolution. We unfortunately cannot find an updated link for the 2016 fact sheet and so have removed it from the references. |
| (in the response to reviewers you incorrectly say that adjusting for clustering would make the results more precise, however I noted that you had correctly stated the effect in the revised paper where you note that failing to adjust for clustering means that “standard errors and p values throughout the analysis may be underestimated”) | Thank you for pointing this out. |
|  |  |
| **Reviewer 3** |  |
| P43, Line12 - Not clear what outcome(s) the sample size calculations are based on? What was the anticipated non-response rate? Was adjusted sample size of 1000 the number to be invited, screened or included? | As this was a survey, the sample size calculation was not done for a specific outcome or hypothesis. There was not an anticipated non-response rate. The sample size of 1000 was the number to be included. |
| P43, Line40 - How were regions selected? i.e., on what basis? | Thank you for raising this. Regions were not selected; LGAs within regions were randomly selected with a probability proportionate to the population aged ≥18. We have corrected this in the manuscript (section 3.1). |
| p48, Line 24 – it would be informative to present the total number of households surveyed (including those without recent medicine use) | We have added this clarification to section 4.1. |
| The authors appear to have used a complete case analysis of their data (i.e., only included participants with complete data) but have not given any indication of how frequently data was missing in their results text or tables. This would help understand the extent to which complete case analysis was appropriate. | Thank you for pointing this out. We have added a footnote on page 10 to clarify that the prevalence of dropped observations due to missing responses was low – less than 5% in most models and never greater than 10%. |
